# Mercury, Lead, Cadmium, Cobalt, Arsenic and Selenium in the Blood of Semipalmated Sandpipers (*Calidris pusilla*) from Suriname, South America: Age-related Differences in Wintering Site and Comparisons with a Stopover Site in New Jersey, USA

**DOI:** 10.3390/toxics6020027

**Published:** 2018-05-09

**Authors:** Joanna Burger, David Mizrahi, Nellie Tsipoura, Christian Jeitner, Michael Gochfeld

**Affiliations:** 1 Division of Life Sciences, Rutgers University, 604 Allison Road, Piscataway, NJ 08854-8082, USA; jeitner@biology.rutgers.edu; 2 Environmental and Occupational Health Sciences Institute (EOHSI), Piscataway, NJ 08854, USA; mg930@eohsi.rutgers.edu; 3 New Jersey Audubon, 11 Hardscrabble Rd, Bernardsville, NJ 07924, USA; david.mizrahi@njaudubon.org (D.M.); nellie.tsipoura@njaudubon.org (N.T.)

**Keywords:** suriname, New Jersey, mercury, selenium, molar ratios, shorebirds, semipalmated sandpiper, temporal patterns

## Abstract

It is essential to understand contaminant exposure and to compare levels of contaminants in organisms at different ages to determine if there is bioaccumulation, and to compare levels encountered in different geographical areas. In this paper, we report levels of mercury, lead, cadmium, cobalt, arsenic and selenium in the blood of semipalmated sandpipers (*Calidris pusilla*) wintering in Suriname as a function of age, and compare them to blood levels in northbound migrants at a stopover in Delaware Bay, New Jersey. We found (1) young birds had higher levels of cadmium, cobalt, and lead than adults (after second year birds); (2) there were no age-related differences for arsenic, mercury and selenium; (3) only four of the possible 16 inter-metal correlations were significant, at the 0.05 level; (4) the highest correlation was between cadmium and lead (Kendall tau = 0.37); and (5) the adult sandpipers had significantly higher levels of cadmium, mercury and selenium in Suriname than in New Jersey, while the New Jersey birds had significantly higher levels of arsenic. Suriname samples were obtained in April, after both age classes had spent the winter in Suriname, which suggests that sandpipers are accumulating higher levels of trace elements in Suriname than in Delaware Bay. The levels of selenium may be within a range of concern for adverse effects, but little is known about adverse effect levels of trace elements in the blood of wild birds.

## 1. Introduction

Environmental scientists, managers, public policy makers, and the public are interested in whether humans and other animals are exposed to increasing levels of contaminants world-wide, especially in developing countries and in remote areas. Most North American shorebird species are declining [1], including semipalmated sandpipers (*Calidris pusilla*), which have declined by approximately 80% since the early 1980s [2]. Exposure to environmental contamination on breeding, stopover, and wintering grounds may be a contributing factor. Assessing levels and trends in wintering sites where few data are available is especially important, both to understand the risk to the species, and to other organisms that might consume them. It is almost impossible to examine contaminant levels in all species, or even in a range of species at different trophic levels. Thus, it is essential to select bioindicators of environmental contamination that provide as much information as possible about global levels, temporal trends, and spatial trends. Bioindicators should be easy to use, easy to understand, indicative of a real change, cost-effective, and amenable to use over a large spatial and temporal scale [3,4]. In addition, sample collection should not adversely affect populations. For birds, feathers are often used for monitoring because they can be obtained non-invasively, stored easily, are easily replaced by the bird, and information on levels exists in the literature [5]. Feather concentrations reflect circulation levels when the feather was growing, which integrates exposure over a period of weeks, and information on molt patterns is essential. Blood levels reflect exposure at the time of collection, and relate directly to levels in other tissues [5,6]. For this reason, and because it can be collected easily from birds, blood is ideal for local monitoring [6].

Migratory shorebirds are useful as bioindicators because they traverse large geographical areas each year, their migratory patterns and molt schedules are known and there is sufficient information on some species to assess age-related and geographical differences. There are few studies of metal levels in shorebirds that breed in the Arctic [7]. Metal levels have been examined in semipalmated sandpipers from Delaware Bay since 1991 [8,9,10,11]. In the Western Hemisphere, many shorebirds breed in the Arctic, and migrate to South America, with some traveling to the southern tip of the continent. During migration they make stops at known, traditional migratory stopover sites. Since the 1980s, Delaware Bay has been recognized as a migratory stopover of “hemispheric importance” in the Western Hemisphere Shorebird Reserve Network. In the 1990s, as many as 270,000 shorebirds were recorded in a single count, and estimates were made of more than 1 million shorebirds using the Bay during spring migration [12]. This represents the largest concentration of northbound shorebirds on the east coast of the United States. During the 2–3 week stopover at Delaware Bay, shorebirds refuel to complete the 4000+ mile journey to the Canadian Arctic and subarctic breeding grounds. Their migration in May coincides with the spawning of horseshoe crabs (*Limulus polyphemus* [13,14]), whose eggs they consume in large quantities as generally they are easy to find, digest, and metabolize [15]. To successfully make the long migration and be ready to mate and lay eggs immediately upon reaching the breeding grounds, shorebirds need to nearly double their weight during the stopover [16]. Doubling their weight in a short period, however, provides an opportunity for a large bolus exposure to contaminants as the birds store fat, which is then released and is metabolized during migration and egg-laying on Arctic breeding grounds.

In this paper, we examine heavy metal levels in the blood of semipalmated sandpipers wintering in Suriname to determine (1) if there are age-related differences; (2) if metal levels are inter-correlated; and (3) if levels are similar in blood from Suriname (wintering) and New Jersey (migrants) sandpipers. Delaware Bay (New Jersey) is a major stopover area for this species on its migration north. We analyzed mercury, cadmium, and lead because they are the major contaminants of concern in marine environments [17,18,19], arsenic because it is a concern for wildlife in marine and estuarine ecosystems [20], and cobalt because it is a contaminant suspected in Suriname due to upstream mining [21]. Some contaminants, such as mercury, were of special interest because they biomagnify up the food chain [22,23]. Mercury is of particular concern because gold mining is the most important mineral extraction, and mercury pollution, both airborne and downstream from alluvial gold mining is a significant environmental problem in Suriname [24].

Examining age-related differences in levels in blood of semipalmated sandpipers from Suriname is also important because the young birds do not migrate north after their first winter, but remain in Suriname throughout their second year. Although juveniles arrive on the wintering grounds later than adults (Mizrahi, Unpubl. data), both age classes had several months to accumulate metal levels in Suriname before they were sampled. First-year birds, however, do not show the same fattening profiles as adults during spring when they are preparing for migration (Mizrahi, Unpubl. data). Significant age-related differences would suggest further studies on potential differences in diet or foraging locations. Understanding correlations among trace elements would suggest a need for controlled laboratory experiments to examine potential synergistic effects.

## 2. Materials and Methods

### 2.1. Collecting Methods

Since 2009, approximately 500 semipalmated sandpipers have been captured each winter along the coast of Suriname, and since the mid-90s, more than 25,000 individuals of the species have been captured as part of an international effort to understand the biology, ecology, and migratory behavior of shorebirds in Delaware Bay. The primary team working in Suriname and Delaware Bay included scientists from New Jersey Audubon, New Jersey Department of Environmental Protection (Endangered and Nongame Species Program), and Conserve Wildlife Foundation. Birds were captured by cannon netting, whoosh netting, and mist netting. Size and weight measurements were recorded, blood was drawn, and birds were marked with uniquely coded leg flags and leg bands. 

Wintering semipalmated sandpipers sampled for this study (*N* = 71) were captured 23–28 April 2013 along the Atlantic coasts at the mouth of Warappakreek, in the Commewijne District, Suriname (5.991275°, −54.913603°). We aged the sandpipers as either young (second-year birds) or adults (all age classes after second-year), based on wing molt stages and plumage characteristics [25]. From each sampled bird we collected approximately 150 microliters of blood in heparinized capillary tubes from the brachial vein. Blood was stored frozen in Eppendorf tubes™ and transported to the Environmental and Occupational Health Sciences Institute of Rutgers University. 

Trace elements were analyzed by atomic absorption, and the levels in the Suriname birds were compared with levels in birds sampled on Delaware Bay, New Jersey, USA (collected in 2011 and 2012, *N* = 30 [11]). Blood levels reflect short-term exposure, depending on ongoing exposure and the biologic half-life of each element. All methods were approved by the Rutgers University Animal Care and Use Committee (92-036), and conformed to guidelines provided by the Ornithological Council (www.nmnh.si.edu/birdnet/guidetouse). These guidelines have been formulated with consideration of animal welfare and research needs.

### 2.2. Chemical and Data Analysis

Blood samples were kept cold (with dry ice) until they were transferred to the Elemental Analysis Laboratory of the Environmental and Occupational Health Sciences Institute at Rutgers University. A 0.2 g (wet weight) sample of blood was digested in 70% Ultrex ultrapure nitric acid and deionized water in a microwave (CEM MDS 2000), using a digestion protocol of three stages of ten min each under 50, 100 and 150 pounds per square inch (3.5, 7, and 10.6 kg/cm^2^) at 70% power. Digested samples were subsequently diluted to 5 mL with deionized water.

Mercury was analyzed by cold vapor atomic absorption spectrophotometry as total mercury, of which about 85–90% was assumed to be methylmercury [26]. Other trace elements were analyzed by graphite furnace (flameless) atomic absorption. Instrument detection limits were 0.02 ppb for arsenic, cadmium and cobalt, 0.15 ppb for lead, 0.2 ppb for mercury and 0.7 ppb for selenium. All results were for whole blood reported in ppb wet weight (1 ppb = 1 ng/g). Blanks, standard reference material (NIST, SRM 1640 “Trace Metals in Natural Water”), and spiked matrix specimens were used to monitor assay performance for all batches. Certified Reference Material (NIST, CRM DORM-2 “Dogfish Muscle Certified for Trace Metals”) was used for mercury. Reference material recoveries ranged from 89% to 101%. All concentrations are expressed in ppb, wet weight. Batches with recoveries of less than 85% were reanalyzed. The coefficient of variation on replicate, spiked samples ranged up to 10%. 

We used non-parametric procedures (Kruskal Wallis test, PROC NPAR1WAY [27]) to determine age and place differences in heavy metal levels, and a non-parametric Kendall test for inter-metal correlations. We used these non-parametric tests because they are more conservative and are best suited for small datasets with no assumption of normality.

## 3. Results

Young birds had significantly higher levels of cadmium, cobalt, and lead than adults (Table 1). Although there were significant differences, they were generally within an order of magnitude. Since selenium is known to partially modify the adverse effects of mercury, Table 1 provides both the selenium/mercury and the mercury/selenium ratios because both are used in the literature. Since selenium levels were high, the selenium/mercury ratio was also high.

Of the 16 possible inter-metal correlations, four were statistically significant (Table 2). The highest correlation was between cadmium and lead, followed by cobalt with cadmium and lead; they were all significant at <0.003 level. Arsenic was weakly associated with mercury and with selenium.

One objective of the study was to determine whether there was a difference between the levels of trace elements in the adult birds collected in Suriname in April (before migration north) and those collected at Delaware Bay (New Jersey) in May (after migration north). The birds from Suriname had significantly higher levels of cadmium mercury, and selenium (Table 3), while those from New Jersey had significantly higher levels of arsenic. The level of significance partly reflects the lack of variation in metal levels in blood at each location.

## 4. Discussion

### 4.1. Age-Related Differences

Exposure to trace elements occurs through food, water, and air, and through ingestion of external contamination on their feathers. For some elements, particularly mercury, bioaccumulation occurs with succeeding levels of the food chain [5,28,29]. In general, levels of trace elements increase with age in birds, but these conclusions usually reflect tissues other than blood [5,30,31,32]. Few authors have examined age-related differences in blood levels of shorebirds, and although Tsipoura et al. [11] examined levels in blood of semipalmated sandpipers from Delaware Bay, they did not age them because most young birds do not migrate north. In the present study, young birds in Suriname had significantly higher levels of cadmium, cobalt, and lead than adults, suggesting greater exposure, greater accumulation, and/or foraging in different habitats on different prey. Contrarily, Riecke et al. [33] found no significant differences in blood lead levels as a function of age in black-necked stilts (*Himantopus mexicanus*) on the upper Texas coast. 

Levels in blood, however, represent mainly current exposure, and not accumulated trace elements, which, subsequent to exposure in blood, are transferred to other tissues where they may be stored [5,11,27]. Thus, the higher levels in young birds represent higher exposure (prey type or quantity) or greater absorption, although the relative rate of transfer of trace elements to other tissues may differ between young and adults. Young birds that hatched the previous summer migrated to South America for their first winter (where they were sampled), then most remained in Suriname over the next year, in contrast to adults that migrated to the Arctic to breed. The blood levels reflect both recent exposure, mainly ingestion, and bioaccumulated trace elements that transfer from tissues to blood. It may be that second year birds do not molt as many feathers as adults while in Suriname, which account for the higher accumulation in young (D. Mizrahi, Unpubl. Data). Further research on the toxicokinetics of movement between blood and other tissues should be examined.

There were no age-related differences in the selenium/mercury molar radios, and the ratios were rather high. Ralston and others [34,35,36] have proposed that selenium protects organisms, including fish and others, from the harmful effects of mercury, and argue that ratios above one are considered protective. However, high levels of selenium are toxic to waterbirds [37], and there is controversy about what ratio can be considered protective, and whether protection is the same for different organs [38].

### 4.2. Locational Differences

Mercury and other chemicals undergo short-range and long-range atmospheric transport, and deposit on land and oceanic environments [39]. Since many industrialized nations poorly regulate mercury and other trace element emissions, atmospheric deposition may be increasing [40]. The rapid increase in illegal alluvial gold mining in Suriname in the past decade has resulted in widespread mercury contamination in soil, water and fish [24]. However, declines over time should be occurring in lead and cadmium because levels have declined in the environment generally from regulations for cadmium in batteries, and the removal of lead from paint and gasoline [41]. Declines in cadmium and lead occurred at the same time, although declines in cadmium were not as great as lead [7,42]. There is insufficient information to know if lead, mercury, and cadmium have declined generally in Suriname.

We expected that there might be locational differences in trace element levels in the blood of semipalmated sandpipers because levels in blood reflect recent, local exposure. With time, trace elements are incorporated into other tissues (feathers, bone, liver, kidneys [23]). The semipalmated sandpiper blood collected in Suriname had significantly higher levels of cadmium, mercury and selenium than samples collected from New Jersey, and New Jersey birds had higher levels of arsenic. Although the differences were not great, they were statistically significant. For these trace elements, the differences suggest that the sandpipers are exposed to higher levels in Suriname, and then as they migrate north to New Jersey and begin to feed on Delaware Bay shores, levels of several trace elements in the blood decline. Normally one thinks of Delaware Bay as being highly contaminated as it is one of the major shipping ports in the U.S., but the data indicate similar or higher levels of most trace elements in blood of sandpipers from Suriname. However, the lack of a large difference suggests that the sandpipers are not picking up unusually high levels of trace elements in either place.

Hargreaves et al. [6,43] examined metal levels in the blood of six species of adult shorebirds from the Arctic (Nunavut, Canada). Mean levels from the Arctic were arsenic (means of non-detect to 11 ppb for white-rumped sandpiper, *Calidris fuscicollis*), cadmium (all non-detect), cobalt (non-detect), lead (means of 4 ppb to 19.0 ppb for black-bellied plover *Pluvialis squatarola*), mercury (means of 20 ppb to 587 ppb for ruddy turnstone *Arenaria interpres*), and selenium (means of 575 to 2550 ppb for red phalarope *Phalaropus fulicarius*). In general, the levels reported by Hargreaves et al. [6] were lower for arsenic, cadmium and lead, and higher for mercury than levels in the semipalmated sandpipers collected in Suriname or in Delaware Bay in the U.S. 

Arsenic was significantly higher in semipalmated sandpipers from Delaware Bay than Suriname. The higher levels likely came from the relatively high levels of arsenic in the eggs of horseshoe crabs, the semipalmated sandpiper’s primary food [44,45,46], and the levels of arsenic in the blood of several shorebirds from Delaware Bay correlate with the levels of arsenic in horseshoe crab eggs [11,23,47].

### 4.3. Effects Levels

Exposure to trace elements and other contaminants can have negative effects on reproduction, egg hatchability, hatchling survivorship, neurobehavioral development and body condition [23,24,47,48,49,50], although there are species-specific differences in effects levels [51]. The metals of primary concern in marine environments are mercury, cadmium and lead [52,53] because they are non-essential, widespread, and highly toxic [54,55]. Marine species can often tolerate higher levels than freshwater species [48,49,56,57], partly because they may detoxify trace elements [58]. Concerns also include local exposure from industries or mining, such as alluvial gold mining in Suriname, and PCBs and PAH exposure from oil transport in Delaware Bay [23].

Much of our understanding of the effects of trace elements on birds has involved examining the effects of mercury and lead in laboratory and field studies [42,59]. Mercury and lead levels in blood associated with adverse reproductive effects in birds are 4000 ppb to 5000 ppb [49,52,60], and in the present study the mean levels of mercury and lead in semipalmated sandpipers were less than 19 ppb (mercury) and 109 ppb (lead), suggesting that these levels are not a cause for concern. 

Cadmium causes more adverse behavioral effects at lower concentrations than lead and mercury [56], including slow growth rates [18]. In this study, the mean cadmium levels in the blood of semipalmated sandpipers averaged 4.4 ppb, well below any effects levels.

Inorganic arsenic can cause muscular incoordination, slowness, falling, hyperactivity and a number of other symptoms in birds, including destruction of blood vessels, shock, and death [61]. Although there have been a number of experiments on the effects of arsenic on birds, these mainly report the exposure dose, and do not report the levels of arsenic in tissues (including blood [61]). The critical levels for fish tissue are 1300 ppb [61,62]. Further, arsenic is known to partly ameliorate the toxic effects of selenium [63]. Arsenic in sandpiper blood in New Jersey was well below that level.

Levels of selenium in feathers known to be associated with effects, range from 1.8 ppm (sublethal) to 26 ppm (lethal), depending upon species [7,64,65], however, adverse effects associated with blood selenium levels have not been investigated. The average level of selenium in blood in semipalmated sandpipers from Suriname was 5590 ppb for adults. There are few studies that have reported the levels of selenium in blood, although levels of selenium in blood from herring gulls (*Larus argentatus*) from a relatively polluted nesting colony in New York were only 350 ppb [66]. The high levels in adults at both sites suggests some potential for adverse effects. Levels of selenium that are associated with adverse effects for shorebirds or other marine birds, however, have not been examined in the laboratory, so interpretation is difficult. 

Elemental selenium and mercury have a high binding affinity and co-precipitate, and are known to modify the toxicity of each other [36,65]. Selenium and mercury levels showed no consistent patterns in the blood of the shorebirds we examined from Suriname and Delaware Bay. This suggests that there may be no consistent relationship in blood, which in turn may suggest no ability to predict if toxicity is ameliorated, but this requires further examination. Other studies in marine birds have reported geographical correlations between mercury and selenium levels, and suggested this is evidence of interactions and protection against toxicity [67]. Indeed, higher mercury levels have been associated with lower reproductive performance in skuas and albatrosses with different impacts reflecting either species sensitivities or spatial variation [68,69]. Mechanisms include the documentation of mercury concentration influencing reproductive hormones in petrels [70].

Much less is known about the levels and effects of cobalt that was examined in blood from the semipalmated sandpipers from Suriname. There are few controlled laboratory studies for cobalt, and those that do, relate adverse effects to liver or kidney levels, making it difficult to interpret the significance of the levels found in the blood of shorebirds. Cobalt levels were generally low, and likely pose no risk.

## 5. Conclusions

The complexities of measuring and interpreting contaminant levels in migratory birds continue to be challenging, with evidence of geographic and taxonomic differences. New techniques for studying migration, diet, behavior, and reproduction, and improved analytic sensitivity and efficiency, are advancing our knowledge, and demonstrating adverse effects that are evident at the population level [67], while identifying the need for controlling contamination at the source. In this study, (1) young semipalmated sandpipers had significantly higher levels of cadmium, cobalt and lead than adults; (2) birds from Suriname had higher levels of cadmium, mercury and selenium than those from New Jersey; (3) New Jersey sandpipers had higher levels of arsenic (although in all cases, the differences were not great); and (4) there were few correlations among trace elements. The levels of all trace elements (except selenium) were well below those reported for blood in other shorebirds, and were below those known to cause adverse effects. The relative importance of selenium in both the prey and tissues of semipalmated sandpipers from Suriname requires further examination to determine potential adverse effects, especially on young birds that remain in the area for over a year.

## Figures and Tables

**Table 1:** Mean (+ standard error) element levels in whole blood collected from semipalmated sandpipers in Suriname, April 2013, and results of comparisons between age classes (second-year versus after second-year). Mean values are given in parts/billion (1 ppb = 1 ng/g). NS = not significant.

Element	Second Year	After Second Year	Age Comparison
	35	36	Kruskal–Wallis (Chi-Square)
	Mean ± SE	Mean ± SE	
Arsenic	222 ± 128	202 ± 88.9	NS
	0.2–590	34–380	
Cadmium	6.2 ± 6.8	2.6 ± 3.3	8.8 (0.003)
	0.1–28	0.1–15	
Cobalt	55.9 ± 78.9	13.2 ± 19.8	10.9 (0.001)
	1–353	0.6–77	
Lead	152 ± 120	67.9 ± 83.9	15.4 (<0.0001)
	22–450	8–500	
Mercury	18.2 ± 11.6	16.8 ± 10.4	NS
	3.4–46.7	1–54	
Selenium	5070 ± 2190	5590 ± 2560	NS
	780–10,000	350–10,000	
Hg/Se Ratio	0.0014:1	0.0012:1	NS
Se/Hg Ratio	707:1	846:1	NS

**Table 2:** Correlations of trace elements in the blood of semipalmated sandpipers from Suriname. (*n* = 71) For all other combinations *p* > 0.10.

Metal			Kendall Tau	*p* <
Arsenic	and	Selenium	0.16	0.05
	and	Mercury	0.14	0.09
Cadmium	and	Lead	0.37	0.0001
	and	Cobalt	0.26	0.003
Cobalt	and	Lead	0.25	0.0003

**Table 3:** Mean element levels in whole blood collected from adult Semipalmated Sandpipers in New Jersey and Suriname. Arithmetic mean values are given in parts per billion (1 ppb = 1 ng/g). NS = not significant.

Element	New Jersey	Suriname	Kruskal–Wallis Chi-Square
	Mean ± SE	Mean ± SE	
Sample Size	30	36	
Arsenic	381 ± 45	202 ± 13.0	11.4 (0.0008)
Cadmium	1.8 ± 0.5	2.6 ± 0.5	3.8 (0.05)
Cobalt	^a^	13 ± 3.3	
Lead	59.8 ± 10.5	68 ± 14.0	NS
Mercury	12.7 ± 3.3	17 ± 1.7	9.2 (0.003)
Selenium	4360 ± 500	5593 ± 427	4.3 (0.04)

^a^ Cobalt was not analyzed in the New Jersey samples.
